# Identification of Differentially Expressed Drought-Responsive Genes in Guar [*Cyamopsis tetragonoloba* (L.) Taub]

**DOI:** 10.1155/2020/4147615

**Published:** 2020-12-03

**Authors:** Aref Alshameri, Fahad Al-Qurainy, Abdel-Rhman Gaafar, Salim Khan, Mohammad Nadeem, Saleh Alansi, Hassan O. Shaikhaldein, Abdalrhaman M. Salih

**Affiliations:** Department of Botany and Microbiology, College of Science, King Saud University, Riyadh 11451, Saudi Arabia

## Abstract

Drought remains one of the most serious environmental stresses because of the continuous reduction in soil moisture, which requires the improvement of crops with features such as drought tolerance. Guar [*Cyamopsis tetragonoloba* (L.) Taub], a forage and industrial crop, is a nonthirsty plant. However, the information on the transcriptome changes that occur under drought stress in guar is very limited; therefore, a gene expression analysis is necessary in this context. Here, we studied the differentially expressed genes (DEGs) in response to drought stress and their metabolic pathways. RNA-Seq via an expectation-maximization algorithm was used to estimate gene abundance. Subsequently, an Empirical Analysis of Digital Gene Expression Data in the R Bioconductor package was used to identify DEGs. Blast2GO, InterProScan, and the Kyoto Encyclopedia of Genes and Genomes were used to explore functional annotation, protein analysis, enzymes, and metabolic pathways. Transcription factors were identified using the PlantTFDB database. Our study identified 499 upregulated and 191 downregulated genes in response to drought stress. Of those, 32 upregulated and six downregulated genes were deemed as novel genes exclusive to guar. An aggregate of 137 protein families, 306 domains, 12 repeats, and two sites were upregulated. The proton-dependent oligopeptide transporter family and transferase, aquaporin transporter, calcium/calmodulin-dependent/calcium-dependent protein kinase, aspartic peptidase A1 family, UDP-glucuronosyl/UDP-glucosyltransferase, and major intrinsic protein were the most upregulated protein families. The upregulated unigenes were associated with 88 enzymes and 77 KEGG pathways. Finally, the MYB-related, MYB, and ERF transcription factor families were upregulated. These data may be useful for understanding the plant molecular response to drought stress.

## 1. Introduction

Drought remains one of the most serious environmental stresses because of the rise in global temperature and a continuous reduction in soil moisture [1]. The dramatic expansion of water-stressed regions requires the improvement of crops with features such as adaptation and drought tolerance through conventional breeding and/or genetic manipulation. In some crops, such as chickpea, improvement via conventional breeding is difficult because of its narrow genetic base. For such crops, gene expression profiling is used to identify stress-responsive pathways and genes [2]. The expression of various genes is induced in plants in response to water limitation. The early response at the cellular level is partly attributed to cell damage and partly corresponds to adaptive processes that cause changes in the metabolism and structure of cells, thus allowing them to operate under water deficit [3]. Currently, a broad spectrum of methods and approaches is being used to recognize stress-responsive genes [4]. *Arabidopsis* is the model plant that is used most frequently to generate data related to drought-responsive gene expression [5–7]. Nevertheless, this information may not be applied to other species because of the wide genetic diversity that occurs in the plant kingdom. Consequently, to comprehend the plant-specific responses to a specific pressure, each plant species should be investigated individually. Many international endeavors have been undertaken over the past decade to improve the research of legumes. Legumes account for roughly one-third of the world's production of seeds and are considered a significant source of plant protein [1]. Two species are commonly used for functional genomic research, *Lotus japonicus* and *Medicago truncatula* [8, 9]. The recent progress in next-generation sequencing (NGS) technologies enabled the bridging of the gap between plant species and model plants. The drought-tolerance mechanisms in plants are extremely complex and highly variable [10]. Despite the fact that drought stress tolerance in guar varies significantly among genotypes, there is limited genomic information on drought stress in this plant. To enhance and develop hardy guar plants with a considerable amount of tolerance, the elucidation of the molecular dynamics of the regulation of the drought stress response is important.

Drought stress induces several biochemical and physiological responses, which are controlled by several genes at the molecular, cellular, and whole-plant level, thus helping the maintenance of water and ionic homeostasis and the protection of the plant from wilting and certain death. This can be accomplished by preserving osmotic interchangeability within the cell, rebuilding the primary and secondary metabolism, and restoring the proteins in their native folded tertiary framework. The drought stress-tolerance mechanisms that have been reported are associated with the accumulation of N-containing metabolites, such as glycine betaine, soluble antioxidant carbohydrates, and proline, which help preserve the cell's fundamental properties [11–14]. Many significant gene groups have been identified, the expression of which is altered in response to drought stress. The most popular among them are those that participate in cellular metabolism, such as the genes involved in cellular transport and signal transduction, genes encoding hydrophilic proteins, and genes encoding heat-soluble proteins [15]. The functional genes encompassed those encoding heat-shock proteins, thus enabling the refolding of proteins and stabilizing polypeptides and membranes under drought stress. The content of abscisic acid (ABA) increases under the stress of drought, which protects plants from instantaneous dehydration via the closing of stoma [16]. Under drought stress, ABA was proven to regulate the expression of several genes [17]. Recent advances in NGS technologies have allowed the mass sequencing of genomes and transcriptomes, thus generating a wide range of genomic data [18]. Studies of genome-wide expression provide breeders with a systematic structure to explain the molecular basis of complex traits.

A considerable amount of studies of leguminous crops have been carried out to clarify the differential gene expression networks under drought conditions. The abundance of 70% of the expressed sequence tags (ESTs) was greater than 2-fold in the water-deficit-tolerant cultivar of chickpea (*Cicer arietinum* L.) at different points of stress treatment [19]. Seventy-five genes in the root tissues of chickpea were assigned to several different transcription factors. For instance, *WRKY*, *bZIP*, and genes encoding zinc-finger-family protein, MYB domain-containing family, a pentatricopeptide repeat-containing protein, and the auxin response factor were identified [20]. A total of 4954 genes were exclusively regulated in the roots of drought-related genotypes of chickpea at the vegetative and reproductive stages [21]. Le et al. [22] identified 3276 upregulated and 3270 downregulated genes in the drought-stressed leaves of soybean (*Glycine max* L.). Many upregulated genes encode the transcription factors (TFs) NAC, AREB, DREB, and ZAT/STZ kinases. Moreover, the downregulation of several genes associated with photosynthesis can serve as an adaptive mechanism for plant survival by contributing to growth delay under drought stress. Shin et al. [23] used contrasting data from two soybean cultivars to establish a classification scheme designed to identify genes that either responded to water deficiencies in both cultivars or had a response from genotype × environment (G × E) interactions. In the roots of soybean, 6,609 genes displayed differential expression modalities in response to different water-deficit stress levels. Genes involved in hormone (auxin/ethylene), carbohydrate, and cell-wall-associated (XTH/lipid/flavonoid/lignin) metabolic pathways were differentially regulated [24]. Moreover, 3498 and 2724 differentially expressed genes (DEGs) were identified in soybean leaf tissues under flooding and drought conditions, respectively. Such genes encompassed those encoding 289 TFs, including bHLH, ERFs, MYB, NAC, and WRKY. Genes associated with chlorophyll synthesis and photosynthesis were downregulated, while genes associated with cell wall synthesis were downregulated under flood stress and upregulated under drought stress [25]. Under adequate irrigation and terminal drought, Wu et al. [26] found 2126 upregulated and 2013 downregulated DEGs in the “Long 22-0579” (drought-tolerant) *Phaseolus vulgaris* genotype. Whereas the Naihua (drought-sensitive) genotype produced much more DEGs (5804 and 1185 up- and downregulated genes, respectively). Under drought stress conditions occurring during flowering and grain filling in the common bean, de Faria Müller et al. [27] found that 802 ESTs were differentially expressed by the tolerant and susceptible genotypes. Drought-sensitive plants were characterized by having approximately twice the number of DEGs than did the tolerant plants of red clover (*Trifolium pratense* L.) after drought. Furthermore, before the onset of drought, the sensitive plants overexpressed several genes that were annotated as being senescence-related, and the concentration of three metabolites, particularly pinitol, but also proline and malate, was increased in their leaves after drought stress [28]. Li et al. [29] explored differential expression in the stems, leaves, and roots of peanut (*Arachis hypogaea*) under water deficit and abscisic acid during three developmental stages (four-leaf, flowering, and pod-formation stages). Those authors reported that 621 genes were rapidly induced under water deficit, 2,665 genes were induced under water deficit + ABA pretreatment, and 279 genes overlapped the water deficit and water deficit + ABA pretreatment conditions. The genes induced under water deficit + ABA pretreatment included 100 TFs, while only 22 putative TFs were induced under water deficit individually. Brasileiro [30] found that the expansin, nitrilase, NAC, and bZIP TFs displayed significant levels of differential expression under drought stress and recovery in two wild *Arachis* species, *Arachis duranensis*, and *Arachis magna*. In *Vicia faba*, Ammar [31] detected 137 upregulated ESTs in the drought-tolerant variety, i.e., Hassawi 2. Among them, 35 ESTs controlled kinases, ion channels, energy production and utilization, and TFs. Such genes included the non-LTR retroelement reverse-associated, ribulose 1,5-bisphosphate carboxylase (*rbcL*) gene, a probable cyclic nucleotide-gated ion channel, polyubiquitin, a potassium channel, calcium-dependent protein kinase, putative respiratory burst oxidase-like protein C, and a novel unigene. A total of 3210 DEGs were detected in the seeds of mung bean (*Vigna radiata* [L.] R. Wilczek) under drought conditions. Genes encoding TFs (MYB, AP2, and NAC), HSPs, embryogenesis abundant (LEA) proteins, methyltransferase, and histones were differentially expressed [32]. An analysis of differential gene expression identified 11,435 and 6,934 up- and downregulated transcripts, respectively, in the seedlings of the drought-tolerant (PDL-2) and drought-sensitive (JL-3) cultivars of lentil (*Lens culinaris* Medikus) [33]. In response to dehydration stress, two landraces of the Bambara groundnut (*Vigna subterranea*) with very similar genotypes showing contrasting transcriptional behaviors. Both genotypes demonstrated a high expression of dehydration-associated genes, even under water-sufficient conditions, suggesting that they evolved to achieve drought tolerance in different ways. Rehydration resulted in a much greater number of DEGs (486 and 391) compared with dehydration stress (189 and 81). Various gene regulators have been identified, such as WRKY40, PRR7, ATAUX2-11, CONSTANS-like 1, MYB60, AGL-83, and zinc-finger proteins, in the DipC and Tiga Nicuru genotypes [34].

However, the information on the transcriptome changes that occur under drought stress in guar is very limited; therefore, an expression analysis in guar genotypes is necessary to narrow down the pathways involved in the drought stress response. Therefore, the “PWP 5595” accession, which has a high tolerance to drought [35], was utilized here. This study was conducted to detect DEGs under drought stress and understand the molecular mechanisms associated with preflowering adaptation to drought stress in guar.

## 2. Materials and Methods

### 2.1. Plant Material

The seeds of the *C. tetragonoloba* drought-tolerant accession “BWP 5595” were planted in pots containing a mixture of 1 : 1 : 1 soil, perlite, and peat moss, then permitted to grow orderly for up to 35 days. The plants were subjected to two treatments with three biological replicates. The control (GC) plants were fully irrigated up to field capacity (FC); in contrast, drought stress-treated plants were irrigated up to 40% of FC. A completely randomized design (CRD) was used to organize the treatments.

### 2.2. Gene Quantification

In our previous works [36, 37], we sequenced and assembled a comprehensive stress-based *de novo* transcriptome for guar, which was used here as a reference transcriptome. The reads were aligned to the reference transcriptome to estimate gene richness using RNA-Seq by Expectation-Maximization (RSEM; [38]). RSEM output data were utilized to construct a count and normalized expression values matrix using the gene-level abundance estimates for all samples.

### 2.3. Quality Check of Conditions and Biological Replicates

The data were examined using the “Perl-to-R” (PtR) script to investigate the correlation and relationship between the drought stress and control conditions. This script was used to create a variety of plots for exploring the matrix of expression data, as follows. First, PtR was run to compare the biological replicates for each condition. Second, PtR was run to generate a correlation matrix for each replicate. Third, a principal components analysis (PCA) was carried out to scout the relationships among the replicates of the treatments (https://github.com/trinityrnaseq/trinityrnaseq/wiki/QC-Samples-and-Biological-Replicates).

### 2.4. Differential Expression Analysis

To identify and cluster DEGs that depended on expression profiles, the Empirical Analysis of Digital Gene Expression Data in R (EdgeR) Bioconductor package [39] was used as mentioned in the following steps. The transcript and gene-length information were collected. DEGs were identified by running a script that performed pairwise comparisons among each of the conditions, including three replicates for each condition. The R package [40] and R studio [41] were used for the analysis and generation of MA and volcano plots. A Trimmed Mean of M values (TMM) normalization [42] followed by expression profiling was carried out. To extract the most differentially expressed genes in response to targeted conditions and to cluster the genes according to their patterns of differential expression throughout the conditions, a script was run using different parameters. The False Discovery Rate (FDR) [43] and fold change (FC) [44] were set at different values to extract the most significant differences. FDR was set at 0.001 and FC was set at 4; then, a modulation of FDR and FC was performed until the most significant genes for the drought stress condition were extracted. Heat maps of drought stress correlation matrix and DE gene vs. drought stress were prepared using the R package and R studio. The DEGs presented in the heat map were split into clusters of genes with similar expression modality at a Ptree of 70, *P* value of 0.001, and FC of 4.

### 2.5. Gene Ontology (GO) and Metabolic Signaling Pathway Analysis

The Blast2GO software suite v4.1 (https://www.blast2go.com/; [45–47]) was used to perform homology searches (BLASTX and BLASTN) with Gene Ontology terms for unique sequence and functional annotation (GO; http://www.geneontology.org/), protein sequence analysis and classification (InterPro, EBI, https://www.ebi.ac.uk/interpro/), enzyme classification (EC) codes, and Kyoto Encyclopedia of Genes and Genomes (KEGG, http://www.genome.jp/kegg/). Sequences were blasted against a nonredundant (nr) protein database that corresponds to the National Center for Biotechnology Information (NCBI, https://www.ncbi.nlm.nih.gov/) via BLASTx-fast. InterPro was performed in parallel with the blasting phase, followed by the Gene Ontology mapping and gene annotation. Subsequently, the Gene Ontology terms that were derived from the BLAST and InterPro steps were merged. Go-slim reduction was applied. BLAST2GO was also used for assigning the genes to cellular processes, biological functions, and cellular components, as well as other salutary statistics.

### 2.6. Identification of Transcription Factors (TFs)

For the identification of TF families, the TF database PlantTFDB v4.0 (http://planttfdb.cbi.pku.edu.cn/download.php; [48]) was used as a reference TF database. Potential TFs in *C. tetragonoloba* were recognized using BLASTx with a cut-off *E* value of 1 × 10^−5^ (Chen & Li, 2017) and best hit in *Arabidopsis thaliana*.

## 3. Results

### 3.1. Quality Check for Drought Stress Treatment and Biological Replicates

The quality control of biological replicates revealed a high homogeneity between the biological replicates for the control (GC) or drought stress (GD) genes (Supplementary Figures 1-8). The biological replicates were more highly correlated within treatments than between them (Supplementary Figure 9 and Supplementary Table 1). A principal component analysis also revealed that the biological replicates were clustered closely according to the type of treatment (Supplementary Figure 10 and Supplementary Table 2), which was very reassuring.

### 3.2. Identification of DEGs

A differential expression analysis calculated the log CPM, log FC, FDR, and *P* value for each gene. Moreover, MA and volcano plots of DEGs are presented (Supplementary Figure 11). The genes that were expressed most differentially (FDR, 0.001; fold-change, 2) were extracted and clustered across the GD and GC groups according to their differential expression patterns. The expression matrix subsets for genes that were upregulated in the GD and GC groups were calculated. All features that exhibited differential expression (DE) in any of the pairwise comparisons were consolidated into a single expression matrix. Supplementary Table 3 displays a Pearson correlation matrix for pairwise sample comparisons dependent on the range of DE genes. Supplementary Figure 12 presents a clustered heat map showing the sample correlation matrix at an FC of 4 and a *P* value of 0.001. The DEGs of GD vs. GC were clustered in a heat map at a *P* value of 0.001 and an FC of 4 (Supplementary Figure 13). Tree cutting of hierarchically clustered genes was performed at the height of 70%, and the genes were partitioned into two clusters with a similar expression modality that represented 499 upregulated and 191 downregulated DEGs in response to drought stress (Supplementary Figure 14). The two resulting clusters of genes were further investigated, as shown in the following steps.

### 3.3. Analysis of Upregulated Drought-Responsive Genes

#### 3.3.1. Blasting, Mapping, and Annotation

A total of 499 upregulated genes were analyzed using BLAST2GO. Among them, 499 genes (100%) underwent InterProScan, 468 (93.6%) were blasted, 386 (77.2%) were mapped, and 378 (75.6%) were annotated. The remaining 32 unblasted genes were deemed as novel genes that were significantly upregulated exclusively in *C. tetragonoloba* in response to drought stress. Such novel genes could be useful for future investigation. The distribution of *E* values revealed that the 9170 hits had an *E* value ≥ 1e^−4^. The significant hits (36.25%) had an *E* value ≥ 1e^−180^, indicating a high hit incidence and a negligible arbitrary background noise. A considerable amount of mapped data (99.35% of genes with mapping information) was obtained from the UniProtKB database (Universal Protein Resource). The remaining small proportion of data was obtained from the Arabidopsis Information Resource (TAIR), the Protein Data Bank (PDB), and the Sol Genomics Network (SGN) (0.38%, 0.26%, and 0.01%, respectively).

#### 3.3.2. Protein Sequence Analysis and Classification (IPS)

**Figure 1:**
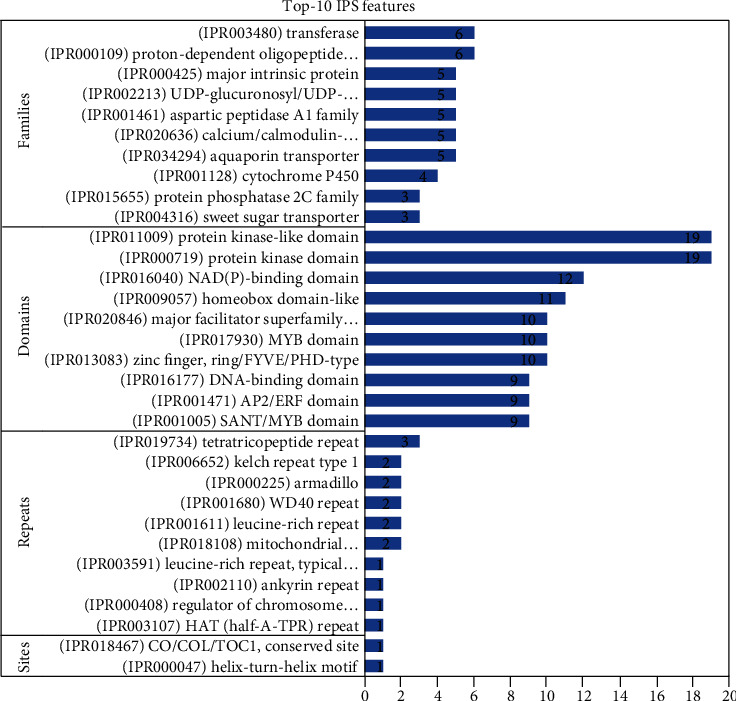
Top 10 upregulated families, domains, repeats, and sites under drought stress.

Out of the 499 genes, 434 (86.8%) had IPS and 283 (65.21%) had GOs. A total of 137 IPS families were found (Figure 1). The “Proton-dependent oligopeptide transporter” family (IPR000109) and the “Transferase” family (IPR003480) comprised an elevated number of genes (six genes), followed by the “Aquaporin transporter” (IPR034294), “Calcium/calmodulin-dependent/calcium-dependent protein kinase” (IPR020636), “Aspartic peptidase A1” (IPR001461), “UDP-glucuronosyl/UDP-glucosyltransferase” (IPR002213), and “Major intrinsic protein” (IPR000425) families, with five genes each. Moreover, an additional 32 families were linked with 2-4 unigenes, and 97 IPS families were linked with a single unigene. A total of 306 domains were detected (Figure 1). “Protein kinase domain” (IPR000719) and “Protein kinase-like domain” (IPR011009) were matched with an elevated number of genes (19 genes for each), followed by “NAD(P)-binding domain” (IPR016040) (12 genes) and “Homeobox domain-like” (IPR009057) (11 genes) and “Zinc finger, RING/FYVE/PHD-type” (IPR013083), “Myb domain” (IPR017930), and “Major facilitator superfamily domain” (IPR020846), with 10 genes each. The remaining 110 domains were matched with 2-9 genes, and 189 domains were matched with single genes.

As reported in Figure 1, a total of 12 repeats were identified. “Tetratricopeptide repeat” (IPR019734) was linked with three genes, followed by “Mitochondrial substrate/solute carrier” (IPR018108), “Leucine-rich repeat” (IPR001611), “WD40 repeat” (IPR001680), “Armadillo” (IPR000225), and “Kelch repeat type 1” (IPR006652), each of which was matched with two genes. In contrast, the remaining six repeats were matched with single genes. Two IPS sites were detected and matched with a single gene: “Helix-turn-helix motif” (IPR000047) and “CO/COL/TOC1, conserved site” (IPR018467).

#### 3.3.3. Functional Annotation

**Figure 2:**
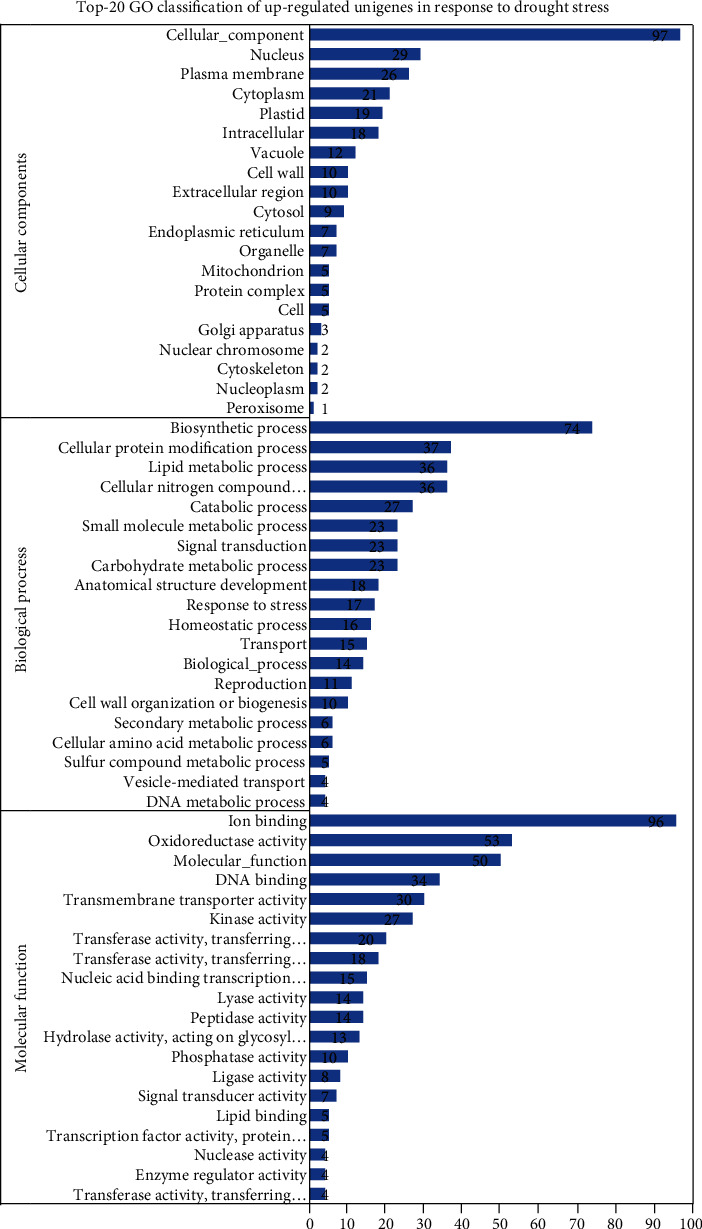
Upregulated GO terms identified in *C. tetragonoloba* under drought stress.

The GO annotations were clustered in three core categories. Biological processes encompassed 37.38% of the overall annotations assigned, while molecular functions and cellular components comprised 37.81% and 24.81% of them, respectively. The GO terms with the greatest number of assigned genes in the category biological process (BP) were: biosynthetic process (74; 16.82%), cellular protein modification process (37; 8.41%), cellular nitrogen compound metabolic process (36; 8.18%), lipid metabolic process (36; 8.18%), and catabolic process (27; 6.14%) (Figure 2). Moreover, the GO terms with the most genes in the cellular component category (CC) were cellular component (97; 33.22%), nucleus (29; 9.93%), plasma membrane (26; 8.90%), cytoplasm (21; 7.19), and plastid (19; 6.51%). Regarding molecular function (MF), the GO terms with the most genes were ion binding (96; 21.57%), oxidoreductase activity (53; 11.91%), molecular function (50; 11.24%), DNA binding (34; 7.64%), transmembrane transporter activity (30; 6.74%), and kinase activity (27; 6.07%).

#### 3.3.4. KEGG Pathway Mapping

**Figure 3:**
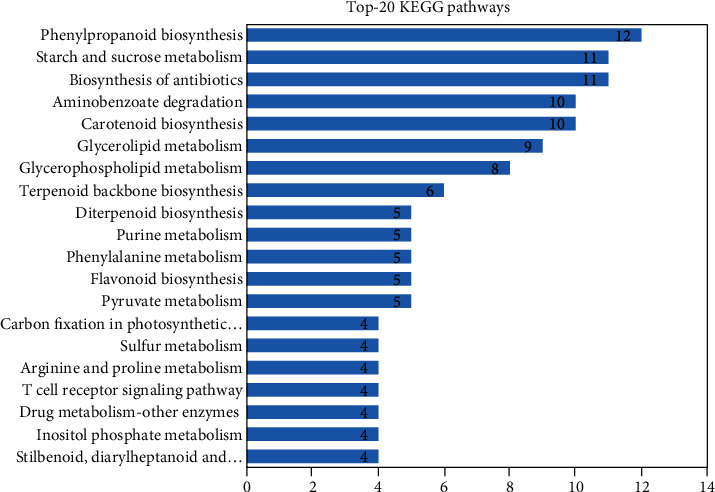
Top 20 upregulated KEGG pathways in *C. tetragonoloba* under drought stress.

The analysis of KEGG pathways revealed that 94 (18.8%) of the 499 genes that were upregulated under drought stress acquired hits in the database of KEGG. These genes were linked with 88 enzymes and 77 pathways (Supplementary Table 4). Among the 77 pathways, the top 20 pathways are presented in Figure 3. The genes linked with Phenylpropanoid biosynthesis (12 genes) were the most representative, followed by Starch and sucrose metabolism (11 genes) and Biosynthesis of antibiotics (11 genes).

**Figure 4:**
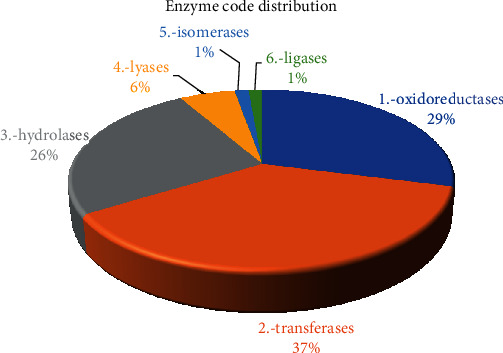
Upregulated enzyme codes (ECs) in *C. tetragonoloba* under drought stress.

Supplementary Figure 15 provides an example of the maps of the pathways. The 88 enzymes were classified into six principal classes. As illustrated in Figure 4, transferase enzymes exhibited an elevated number of genes (54; 37.24%), followed by oxidoreductases (42; 28.97), hydrolases (37; 25.52), lyases (8; 5.52), and both isomerases and ligases, which showed the lowest count of genes (1; 1.38% for each).

#### 3.3.5. Upregulated Transcription Factors

**Figure 5:**
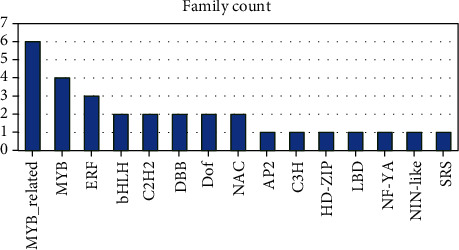
Distribution of the TFs that were upregulated in *C. tetragonoloba* in response to drought stress.

A total of 30 upregulated TFs linked with upregulated DEGs were identified here. These upregulated TFs belonged to 15 families. The MYB-related family involved the most TFs (six TFs), followed by MYB (four TFs) and ERF (three TFs). Other families involved two or one TFs (Figure 5). The summary of the best hits of these TFs against *A. thaliana* is provided in Supplementary Table 5.

### 3.4. Analysis of Downregulated Drought-Responsive Genes

#### 3.4.1. Blasting, Mapping, and Annotation

A total of 192 downregulated genes were analyzed using BLAST2GO. Of these genes, 192 (100%) underwent InterProScan, 186 (96.88%) were blasted, 150 (87.13%) were mapped, and 92 (47.92.6%) were annotated. The remaining six unblasted genes were deemed as novel genes that were significantly downregulated exclusively in *C. tetragonoloba* in response to drought stress. Such novel genes could be useful for future investigation. The distribution of *E*- values revealed that the 3687 hits had an *E* value ≥ 1e^−4^. The significant hits (29%) had an *E* value ≥ 1e^−180^, indicating a high hit incidence and a negligible arbitrary background noise. A considerable amount of mapped data (99.53% of the genes with mapping information) was obtained from the Universal Protein Resource (UniProtKB) database. The remaining small portion of data was obtained from the Arabidopsis Information Resource (TAIR) and the Protein Data Bank (PDB) (0.25% and 0.22%, respectively).

#### 3.4.2. Protein Sequence Analysis and Classification (IPS)

**Figure 6:**
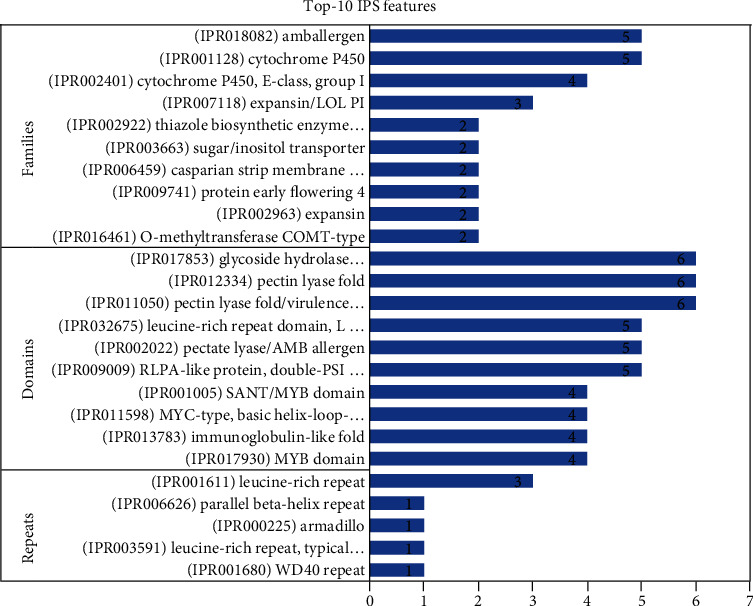
Top 10 downregulated families, domains, repeats, and sites under drought stress.

Out of 192 genes, there were 168 (87.5%) had IPS and 92 (54.8%) of them had GOs. A total of 65 IPS families were found. The “Cytochrome P450” (IPR001128) and “AmbAllergen” (IPR018082) families had an elevated number of genes (five genes), followed by “Cytochrome P450, E-class, group I” (IPR002401) (four genes) and “Expansin/Lol pI” (IPR007118) (three genes). Moreover, an additional nine families were related to two unigenes, and 52 IPS families were related to single genes. A total of 133 domains were detected (Figure 6). “Pectin lyase fold/virulence factor” (IPR011050), “Pectin lyase fold” (IPR012334), and “Glycoside hydrolase superfamily” (IPR017853) were matched with an elevated number of genes (six genes for each), followed by “RlpA-like protein, double-psi beta-barrel domain” (IPR009009), “Pectatelyase/Amb allergen” (IPR002022), and “Leucine-rich repeat domain, L domain-like” (IPR032675), with five genes for each. The remaining 35 domains were matched with 2-4 genes, and 92 domains were matched with single genes.

As reported in Figure 6, a total of five repeats were identified. “Leucine-rich repeat” (IPR001611) was matched with three genes, followed by “WD40 repeat” (IPR001680), “Leucine-rich repeat, typical subtype” (IPR003591), “Armadillo” (IPR000225), and “Parallel beta-helix repeat” (IPR006626), each of which was matched with a single unigene. Surprisingly, no IPS sites were detected.

#### 3.4.3. Functional Annotation

**Figure 7:**
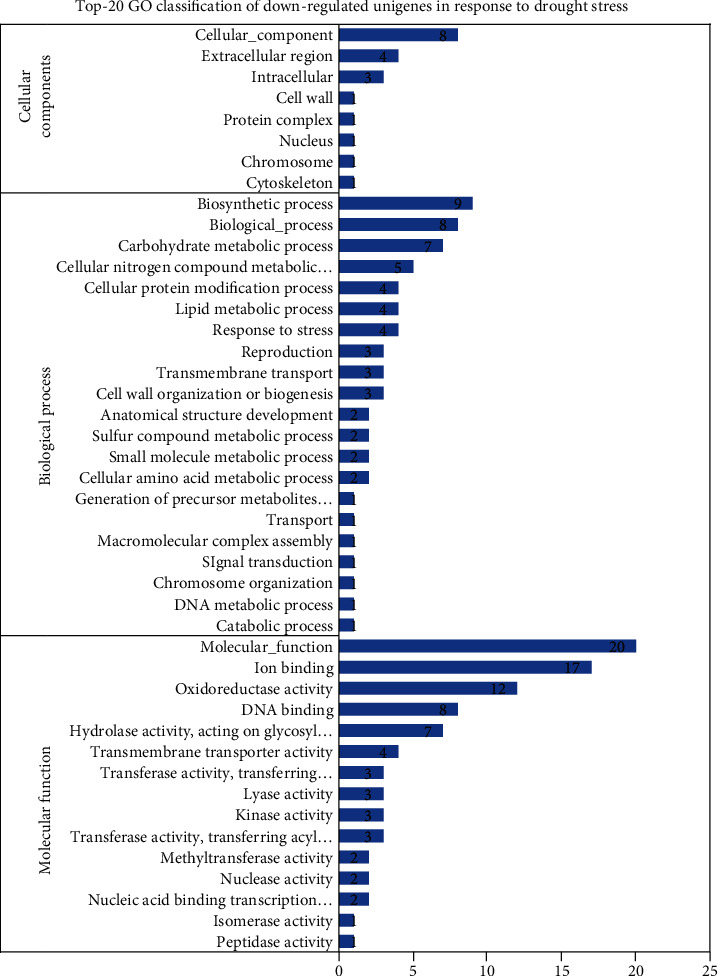
Downregulated GO terms identified in *C. tetragonoloba* under drought stress.

Biological processes encompassed 37.57% of the overall annotations assigned, whereas molecular functions and cellular components comprised 50.87% and 20% of them, respectively. The GO terms with the greatest number of assigned genes in the BP category were biosynthetic process (nine; 13.85%), biological process (eight; 12.31%), carbohydrate metabolic process (seven; 10.77%), cellular nitrogen compound metabolic process (five; 7.69%), and stress responsive (four; 6.15%) (Figure 7). Moreover, the terms with the most genes in the CC category were cellular component (eight; 40%), extracellular region (four; 20%), intracellular (three; 15%), cytoskeleton (one; 5%), and chromosome (one; 5%). In the MF category, the terms with the greatest number of significant genes were molecular function (20; 22.73%), ion binding (17; 19.32%), oxidoreductase activity (12; 13.64%), DNA binding (eight; 9.09%), hydrolase activity, acting on glycosyl bonds (seven; 7.95%), transmembrane transporter activity (four; 4.55%), and transferase activity, transferring acyl groups (three; 3.41%).

#### 3.4.4. KEGG Pathway Mapping

**Figure 8:**
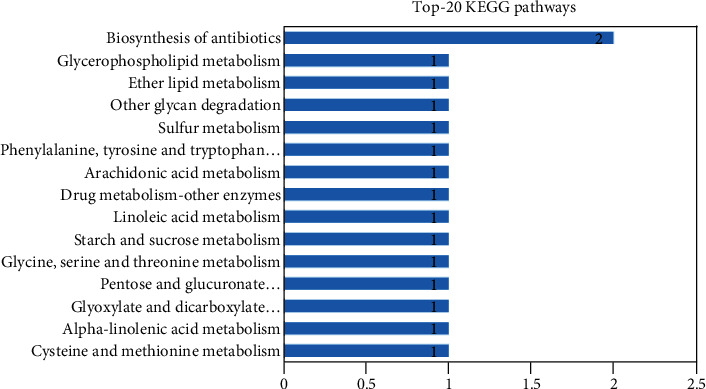
Top 20 downregulated KEGG pathways in *C. tetragonoloba* under drought stress.

The analysis of KEGG pathways revealed that seven (3.64%) of the 192 genes that were downregulated under drought stress acquired hits in the KEGG database. These genes were linked with eight enzymes and 15 pathways (Supplementary Table 6). Of the 15 pathways, the top pathways are presented in Figure 8. Two genes were linked with the biosynthesis of antibiotics pathway, whereas the remaining pathways were linked with single genes.

**Figure 9:**
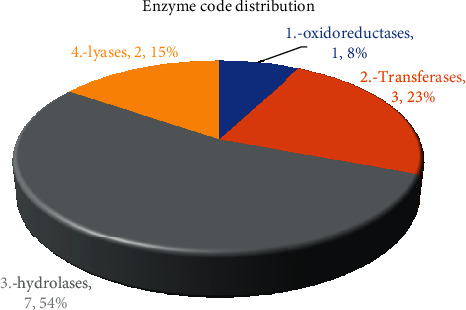
Downregulated enzyme codes (ECs) in *C. tetragonoloba* under drought stress.

The enzymes were classified into four principal classes. As illustrated in Figure 9, hydrolase enzymes exhibited an elevated number of genes (seven; 54%), followed by transferases (three; 23%), lyases (two; 15), and oxidoreductases (one; 8%).

#### 3.4.5. Downregulated TFs

**Figure 10:**
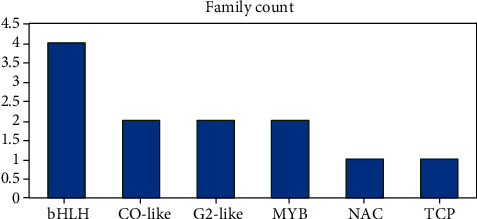
Distribution of downregulated TFs in response to drought stress.

A total of 12 downregulated TFs linked with DEGs were identified. These downregulated TFs belonged to six families. The bHLH-related family involved the greatest number of TFs (four). The remaining families involved two or one TFs (Figure 10). The summary of the best hits of these TFs against *A. thaliana* is provided in Supplementary Table 7.

## 4. Discussion

Drought is the main environmental stress factor affecting the growth of cultivated plants. Moreover, this effect is reinforced by global warming, water shortage, demographic growth, and environmental degradation [49]. The impact of drought is determined based on the severity of the stress and the ability of plants to adapt to this stress, which is determined by physiological, biochemical, and molecular processes [50]. Because plants are susceptible to various forms of water stress, researchers have used experimental conditions ranging from moderate water stress over the full plant cycle [51] to severe stress over short periods [52].

In this investigation, we studied the responsive genes to drought stress (40% of field capacity) at the preflowering stage using the leaves of the drought-tolerant guar accession “PWP 5595” compared with the control condition (field capacity). An RNA-seq approach and next-generation sequencing technology were used in this investigation. A differential expression analysis showed that cutting the hierarchically clustered gene tree at a 70% height resulted in the division of genes into two main clusters: 499 upregulated DEGs and 191 downregulated DEGs. Our findings fell in the range of preceding studies of drought stress-responsive DEGs. For example, the range of the number of DEGs in this context varied from 81 [34] to 18369 [33]. The distribution of *E* values revealed that the hits had an *E* value ≥ 1e^−4^ and that the greatest number of hits (36% and 29% of upregulated and downregulated DEGs, respectively) had an *E* value ≥ 1e^−180^, indicating a high hit rate and a negligible arbitrary background noise. The purpose of UniProt is to offer a complete, high-quality, and freely accessible protein sequence and functional knowledge to the research community. A highly significant amount of our mapping data (99.35% and 99.53% of up- and downregulated DEGs, respectively) was derived from the UniProtKB database. Gene Ontology (GO) describes the definitions/classes that are used to explain gene function and the relationship between those concepts. It categorizes roles based on three aspects: molecular function, cellular component, and biological process [53]. A total of 378 (75.6%) upregulated and 92 (47.92.6%) downregulated DEGs were assigned a GO classification. These findings are in alignment with those of de Faria Müller [27], who reported that 73% of the putative genes were assigned GO terms. Of the three core GO annotation categories, MF comprised the majority of the overall annotations assigned to up- and downregulated DEGs (37.81% and 50.87%, respectively), followed by biological processes (37.38% and 37.57%) and cellular components (24.81% and 20%). These outcomes are similar to those reported by Li et al. [29]. The GO terms with the greatest number of assigned genes in the BP category were biosynthetic process, cellular protein modification process, cellular nitrogen compound metabolic process, lipid metabolic process, and catabolic process. In turn, within the CC category, the terms with the most genes were cellular component, nucleus, plasma membrane, cytoplasm, and plastid. In the MF category, the terms with the most genes were ion binding, oxidoreductase activity, molecular function, DNA binding, transmembrane transporter activity, and kinase activity. These results are partially in keeping with those of previous studies [26, 27, 32, 54]. InterPro analyzes proteins functionally by categorizing them into families and anticipating domains and interesting sites. We blasted our set of drought-responsive DEGs to the 14 databases of the InterPro consortium. In the upregulated DEG collection, we found 137 protein families, 306 domains, 12 repeats, and two sites. In turn, our downregulated DEGs were matched with 65 families, 133 domains, and five repeats.

The proton-dependent oligopeptide transporter (POT) family consists of several energy-dependent transporters found in organisms as diverse as bacteria and humans [55]. They seem to be mainly involved in the intake of small peptides [56]. However, some family members are nitrate permeates, while others are involved in histidine transport [57]. The nitrate transporter AtNRT1.1 (CHL1) works in stomatal opening and improves drought tolerance in *Arabidopsis* [58]. Moreover, AtTGA4, a bZIP transcription factor, provides drought resistance by increasing the transport and assimilation of nitrates in *A. thaliana*. Gu et al. [59] uncovered a correlation between NRT2.1 expression, NO_3_^−^ use, and drought stress and raised the exciting possibility that NRT2.1 plays an important role in the resistance to drought. In the current study, we reported six upregulated DEGs coded to the “Proton-dependent oligopeptide transporter family,” which supports the findings of previous studies.

The large and diverse family of intrinsic proteins (MIPs) comprises more than 100 members that belong to transmembrane channels [60]. There are three subfamilies in the MIP superfamily: aquaglyceroporins, S-aquaporins, and aquaporins [61]. Aquaporins (AQPs) are water selective, whereas the aquaglyceroporins are permeable to water and other small uncharged molecules, such as glycerol. In contrast, the third subfamily contains superaquaporins (S-aquaporins), with little conserved amino acid sequences around the NPA boxes. Plant tonoplast intrinsic proteins (TIPs) are a family that includes water stress-induced isoforms (WSIs). Such proteins can enable water, peptides, and/or amino acids to diffuse from the inner side of the tonoplast to the cytoplasm. Previous studies focused on the role of MIP family members in drought tolerance. For instance, *GmTIP2;1* was recognized as a putative candidate gene implicated in the drought response. Overexpression of the tomato *SlTIP2;2* gene in transgenic tomato plants resulted in increased drought tolerance because of the ability of plants to regulate their transpiration rate under drought stress conditions [62]. The overexpression of GmTIP2;1 improved drought and salt tolerance in *Glycine soja* [63]. In addition, the *Glycine max* plasma membrane intrinsic protein (*GmPIP2;9*) was significantly upregulated under drought stress treatment. In turn, *GmPIP2;9* overexpression increased drought stress tolerance [64]. Moreover, the protein products of the plasma membrane intrinsic *SlPIP2;1*, *SlPIP2;*7, and *SlPIP2;5* aquaporin genes conferred improved tolerance to Tomato against drought stress [65]. In our study, five upregulated DEGs were linked to the MIP family and the aquaporin transporter family. The description of these genes in the Gene Ontology included probable aquaporin TIP-type alpha, probable aquaporin TIP2-2, aquaporin NIP6-1-like, aquaporin NIP6-1, and aquaporin NIP2-1-like.

UDP-glucuronosyl/UDP-glucosyltransferase is a superfamily of enzymes that catalyze the binding of the glycosyl group to a small hydrophobic molecule from UTP-sugar. The *Arabidopsis* uridine diphosphate (UDP)-glycosyltransferase 76C2 (UGT76C2) is known as a cytokinin glycosyltransferase. The results reported by Li et al. [66] showed that UGT76C2, as a cytokinin glycosyltransferase, is implicated in plants' response to water deficit and could be novel in the adaptation to abiotic stress. The *Arabidopsis* UDP-glycosyltransferases UGT79B2 and UGT79B3 also contribute to the tolerance to drought, cold, and salt stresses by modulating anthocyanin accumulation [67]. In our study, five upregulated DEGs were coded to the UDP-glucuronosyl/UDP-glucosyltransferase family, including the UDP-glucose flavonoid 3-*O*-glucosyltransferase, the UDP-glucose flavonoid 3-*O*-glucosyltransferase 7-like, the UDP-glucosyltransferase family, and the UDP-glycosyltransferase 73C3-like.

Aspartic peptidases are well-known proteolytic enzymes in various species, including plants [68]. Overexpression of the aspartic protease in guard cell 1 (*ASPG1*) gene confers drought avoidance in *Arabidopsis* [69]. PCS1 acts in deciding the cell fate throughout reproductive procedures and in the development of embryos in *Arabidopsis*; PCS1 overexpression leads to male sterility by preventing anther dehiscence, while the loss of its function results in excessive cell death during embryogenesis and gametogenesis [70]. Five upregulated DEGs in our study were coded to the Aspartic Peptidase A1 family, including aspartic proteinase PCS1-like, aspartic proteinase Asp1-like, aspartic protease in guard cell 1, and aspartic proteinase nepenthesin-1.

In eukaryotic signal transduction, calcium ions have been recognized as a major conserved second messenger. In plants, the multigene family of CDPKs encodes structurally preserved, unimolecular calcium sensor/protein kinase effector proteins. CDPKs have been known for many years to participate in the Ca^2+^-related signal transduction induced by abiotic stress stimuli in the context of salinity, drought, and cold [71]. A positive regulatory effect of CDPKs in drought stress signaling was explained by the improved expression of ABA-responsive genes [72]. Ciesla et al. [73] reported that the gene encoding the calcium-dependent protein kinase HvCPK2a was significantly upregulated in barley (*Hordeum vulgare* L.) in response to drought. The CBL-interacting protein kinase 16 gene was differentially expressed in three barley genotypes throughout the reproductive stage under drought stress [74]. Five upregulated DEGs in our study were coded to the calcium/calmodulin-dependent/calcium-dependent protein kinase family, including CBL-interacting serine threonine-kinase 12-like, CBL-interacting serine threonine-kinase 14-like, CBL-interacting kinase 2-like, and CBL-interacting serine threonine-kinase 10-like.

In plants, cytochrome P450s represent about 1% of the protein-coding sequences and constitute by far the largest family of enzymes involved in plant metabolism, including hormone biosynthesis and catabolism, as well as the synthesis of primary and secondary metabolites [75]. A nonsynonymous point mutation that is a part of a *P450* gene cluster causes the DSS1 rice mutant. In line with the accumulation of ABA and metabolites, germination and early growth in DSS1 were postponed, which also displayed increased drought tolerance [76]. In tobacco, the *cytochrome P450 CYP94C1* gene was upregulated after 40 min in the roots, whereas in leaves upregulated later with lower foldchange [77]. The overexpression of *SoCYP85A1*, a spinach cytochrome p450 gene, in transgenic tobacco improved root growth and drought tolerance [78]. In our study, four upregulated DEGs were linked to the cytochrome P450 family, including cytochrome P450 72A15-like, cytochrome P450 78A5-like, abscisic acid 8-hydroxylase 1-like isoform X2, and abscisic acid 8-hydroxylase 4. Conversely, nine downregulated DEGs were coded to the cytochrome P450 and cytochrome P450, E-class, group I families, including cytochrome P450, cytochrome P450 71D11, cytochrome P450 83B1-like, cytochrome P450 85A, and cytochrome P450 85A-like.

Type 2C protein phosphatases (PP2Cs) are preserved evolutionarily from prokaryotes to eukaryotes and play a significant role in stress signaling. In *A. thaliana*, the ABA sensor RCAR and PP2Cs such as ABI1 and ABI2 form the holoreceptor for the ABA stress signal induced under water-deficit conditions [79]. In our study, three upregulated DEGs were coded to the protein phosphatase 2C family, including probable phosphatase 2C 51, probable phosphatase 2C 6, and probable phosphatase 2C 33.

The SWEET sugar transporter family contains specific sugar outflow transporters that are essential for plant nectar production and plant seed and pollen development, which are important for the viability of pollen in *Arabidopsis* [80]. The *Arabidopsis* tonoplast monosaccharide transporter (TMT) family comprises three members that are articulated in a tissue- and cell-specific manner. Under drought and cold stresses, the expression of TMT1 and TMT2 is upregulated, indicating a role for these transporters in the response to abiotic stress [81]. Processes that consume sucrose are susceptible to stress inhibition. The accumulation of sucrose, for example, is observed in drought-exposed plants [82]. The bidirectional sugar transporter-encoding *SWEET12* gene is differentially upregulated under drought stress in potato (*Solanum tuberosum*) [83]. In our study, three upregulated genes were coded to the SWEET sugar transporter family, including bidirectional sugar transporter, SWEET12-like, bidirectional sugar transporter, SWEET2-like, and bidirectional sugar transporter N3-like.

TFs are proteins that mediate the transcription of DNA into RNA. They include many proteins, with the exception of RNA polymerase, that initiate and control gene transcription. TFs have DNA-binding domains that give them the ability to bind to particular DNA sequences called enhancer or promoter sequences. Some TFs bind to a DNA promoter sequence near the transcription start site and help form the transcription initiation complex. Many TFs bind to regulatory sequences, such as enhancer sequences, and can either stimulate or suppress the transcription of the associated gene. In our study, 30 and 12 up- and downregulated DEGs were linked with TFs, respectively.

TFs of the MYB family play paramount roles in the growth of plants and in abiotic responses. For instance, OsMYB48-1 overexpression in rice greatly increased tolerance to simulated drought. These results suggest that OsMYB48-1 acts as an uncouth MYB-related TF, which plays a helpful role in the tolerance to drought by controlling stress-induced ABA synthesis [84]. Overexpression of OsMYBR1 provides increased drought tolerance and decreased ABA responsiveness in rice [85]. *A. thaliana* transgenic plants with OsMYB3R-2 overexpression display increased tolerance to drought, salt, and cold stresses. The expression of *dehydration*-*responsive element*-*binding protein 2A*, *COR15a*, and *RCI2A* was increased in *OsMYB3R*-*2*-overexpressing plants compared with the wild-type counterparts, suggesting *OsMYB3R*-*2* as a master switch in stress tolerance [86]. A wheat R2R3-MYB gene, TaMYB30-B, enhances tolerance to drought in transgenic *Arabidopsis* [87]. AtMYB15 overexpression leads to increased tolerance to drought and susceptibility to ABA in *A. thaliana* [88]. TaMYB33 overexpression improves tolerance to drought and salt in *Arabidopsis* [89]. Overexpression of the maize *MYB48* gene confers tolerance to drought in transgenic *Arabidopsis* plants [90]. OsMYB55 expression in maize stimulates stress-reactive genes and increases the tolerance to drought and heat [91]. Transgenic *Arabidopsis* overexpressing AtMYB44 exhibits a remarkably improved drought and salt stress tolerance compared with wild-type plants [92]. In our study, 10 upregulated DEGs were coded to MYB and MYB-related TFs, including LHY-like, LHY-like isoform X1, myb-related 306-like, myb-related Myb4-like, REVEILLE 2-like, REVEILLE 7-like, REVEILLE 8-like isoform X1, and transcription factor MYB44-like.

The members of the AP2/ERF TF family share a well-conserved DNA-binding domain. Although ethylene response factors (ERFs) are usually regarded as mediators of ethylene-related reactions, including members who respond to drought stress [93, 94] and can confer tolerance to these stresses via their overexpression in transgenic plants [94]. This TF family involves DRE-binding proteins (DREBs) that enable the expression of abiotic stress-responsive genes by specifically binding to the dehydration-responsive element/C-repeat (DRE/CRT) [95]. Overexpression of DREB1s/CBFs stimulates the downstream expression of stress-responsive genes and increases tolerance to drought in *Arabidopsis* [96]. Overexpression of a constitutively active form of DREB2A induces the expression of dehydration genes and improves drought tolerance in *Arabidopsis* [97]. Transgenic *Arabidopsis* overexpressing *DREB1D/CBF4* exhibited expression of dehydration-inducible DREB1/CBF target genes and drought tolerance [98]. In our study, three upregulated DEGs were linked with the ERF family, including the ethylene response factor, ethylene-responsive transcription factor At4g13040 isoform X1, and ethylene-responsive transcription factor ERF039-like. These TFs can participate in the regulation of gene expression by stress factors and components of stress signal transduction pathways.

## Data Availability

The raw sequence data has been deposited at the NCBI Short Read Archive (SRA) with accession numbers (SRR10120602, SRR10120603, SRR10120604, SRR10120608, SRR10120611, and SRR10120612).

## Supplementary Materials

Supplementary Materials Supplementary Figure 1: pairwise comparisons of replicate log(CPM) values (data points more than 2-fold different are highlighted in red) in the control condition (GC). Supplementary Figure 2: pairwise MA plots (*x*-axis: mean log(CPM), *y*-axis: log(fold-change)) in the control condition (GC). Supplementary Figure 3: total mapped putative genes in the control condition (GC). Supplementary Figure 4: heat map of the Pearson correlation replication in the control condition (GC). Supplementary Figure 5: pairwise comparisons of replicate log(CPM) values (data points more than 2-fold different are highlighted in red) in the drought stress condition (GD). Supplementary Figure 6: pairwise MA plots (*x*-axis: mean log(CPM), *y*-axis: log(fold_change)) in the drought stress condition (GD). Supplementary Figure 7: total mapped putative genes in the drought stress condition (GD). Supplementary Figure 8: heat map of the Pearson correlation replication in the drought stress condition (GD). Supplementary Figure 9: correlation matrix between the drought stress (GD) and the control (GC) conditions. Supplementary Figure 10: principal component analysis (PCA) of the drought stress (GD) and control (GC) conditions. Supplementary Figure 11: distribution of genes according to fold change (FC), counts, and FDR. Significant DEGs as classified by EdgeR (red) typically showed relatively high read counts associated with low gene expression. Supplementary Figure 12: clustered heat map illustrating the correlation matrix of DEGs between the control (GC) and drought stress (GD) conditions at an FC of 4 and a *P* value of 0.001. Supplementary Figure 13: DEG clustered heat map of control (GC) vs. drought stress (GD) conditions at an FC of 4 and a *P* value of 0.001. Supplementary Figure 14: expression modalities of the DEGs in each cluster under the control and drought stress conditions. Mean expression profile for the cluster (blue) and gene plots (gray). Supplementary Figure 15: the pathway of Phenylpropanoid biosynthesis was upregulated under drought stress. Supplementary Table 1: values of the principal component analysis for the biological replicates of the drought stress (GD) and control (GC) conditions. Supplementary Table 2: values of the Pearson correlation between the biological replicates of the drought stress (GD) and control (GC) conditions. Supplementary Table 3: values of the Pearson correlation matrix between the biological replicates of the drought stress (GD) and control (GC) conditions based on the set of DEGs. Supplementary Table 4: list of KEGG pathways that were upregulated in response to drought stress. Supplementary Table 5: best hit of the transcription factors (TFs) that were upregulated in *A. thaliana*. Supplementary Table 6: list of the KEGG pathways that were downregulated in response to drought stress. Supplementary Table 7: best hit of the transcription factors (TFs) that were downregulated in *A. thaliana*.
