# Supplementary material for: Identification of Differentially Expressed Drought-Responsive Genes in Guar [Cyamopsis tetragonoloba (L.) Taub]
Source: Int J Genomics. 2020 Dec 3;2020:4147615. doi: 10.1155/2020/4147615 (PMC7732403; doi:10.1155/2020/4147615)
Supplement: Supplementary Materials — Supplementary Figure 1: pairwise comparisons of replicate log(CPM) values (data points more than 2-fold different are highlighted in red) in the control condition (GC). Supplementary Figure 2: pairwise MA plots (x-axis: mean log(CPM), y-axis: log(fold-change)) in the control condition (GC). Supplementary Figure 3: total mapped putative genes in the control condition (GC). Supplementary Figure 4: heat map of the Pearson correlation replication in the control condition (GC). Supplementary Figure 5: pairwise comparisons of replicate log(CPM) values (data points more than 2-fold different are highlighted in red) in the drought stress condition (GD). Supplementary Figure 6: pairwise MA plots (x-axis: mean log(CPM), y-axis: log(fold_change)) in the drought stress condition (GD). Supplementary Figure 7: total mapped putative genes in the drought stress condition (GD). Supplementary Figure 8: heat map of the Pearson correlation replication in the drought stress condition (GD). Supplementary Figure 9: correlation matrix between the drought stress (GD) and the control (GC) conditions. Supplementary Figure 10: principal component analysis (PCA) of the drought stress (GD) and control (GC) conditions. Supplementary Figure 11: distribution of genes according to fold change (FC), counts, and FDR. Significant DEGs as classified by EdgeR (red) typically showed relatively high read counts associated with low gene expression. Supplementary Figure 12: clustered heat map illustrating the correlation matrix of DEGs between the control (GC) and drought stress (GD) conditions at an FC of 4 and a P value of 0.001. Supplementary Figure 13: DEG clustered heat map of control (GC) vs. drought stress (GD) conditions at an FC of 4 and a P value of 0.001. Supplementary Figure 14: expression modalities of the DEGs in each cluster under the control and drought stress conditions. Mean expression profile for the cluster (blue) and gene plots (gray). Supplementary Figure 15: the pathway of Phenylpropan [file 4147615.f1.docx]

**
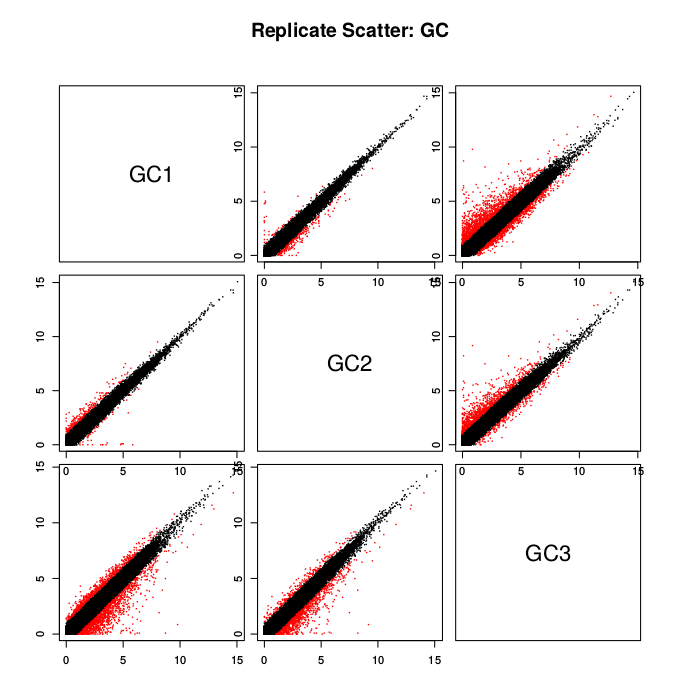
**

**Supplementary Figure 1:** Pairwise comparisons of replicate log(CPM) values (data points more than 2-fold different are highlighted in red) in the control (GC).

**
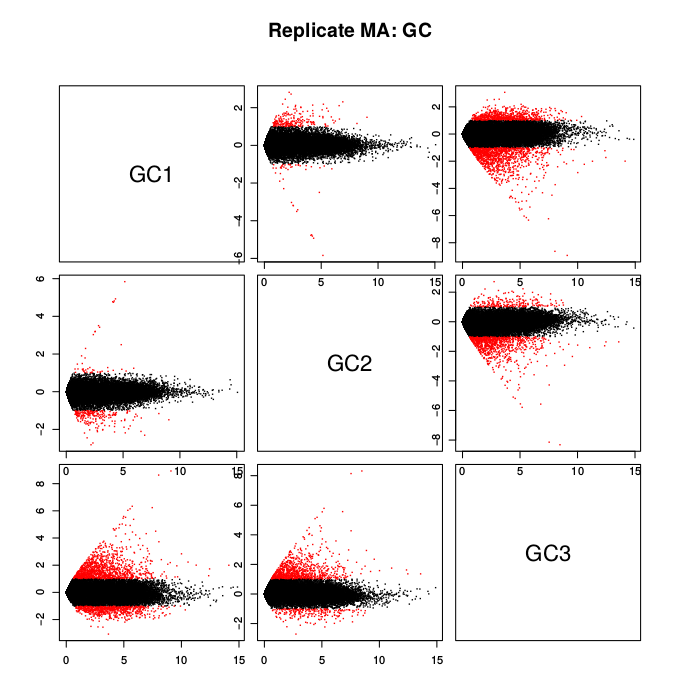
**

**Supplementary Figure 2:** Pairwise MA plots (x-axis: mean log(CPM), y-axis log(fold_change)) in control (GC).


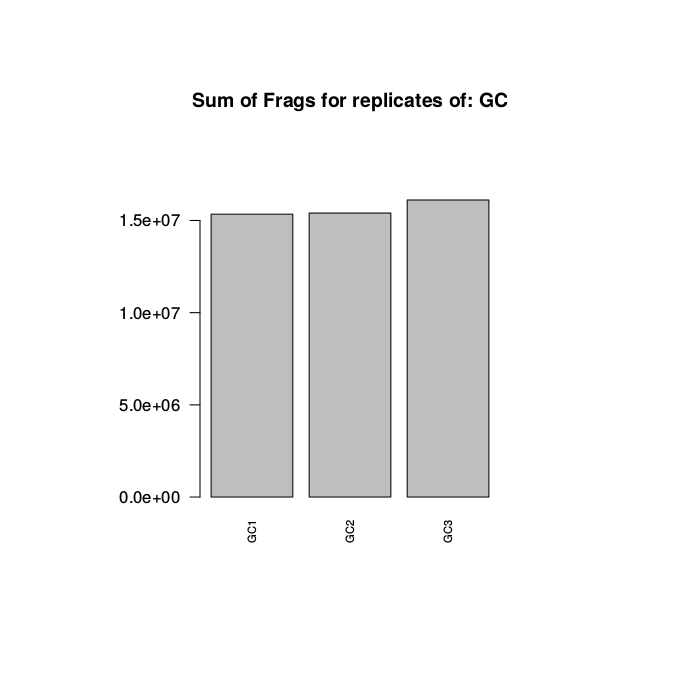


**Supplementary Figure 3:** The total of mapped putative genes in the control (GC).


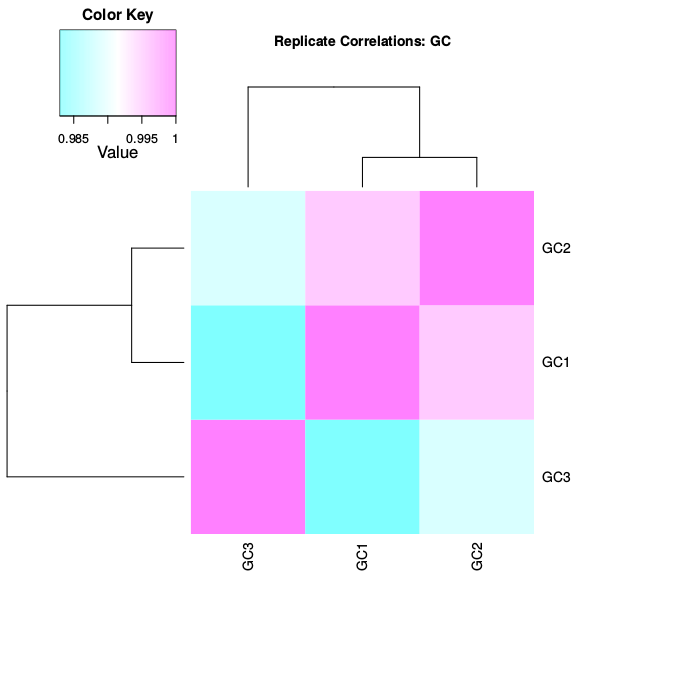


**Supplementary Figure 4:** The heatmap of Pearson correlation replication in the control (GC).


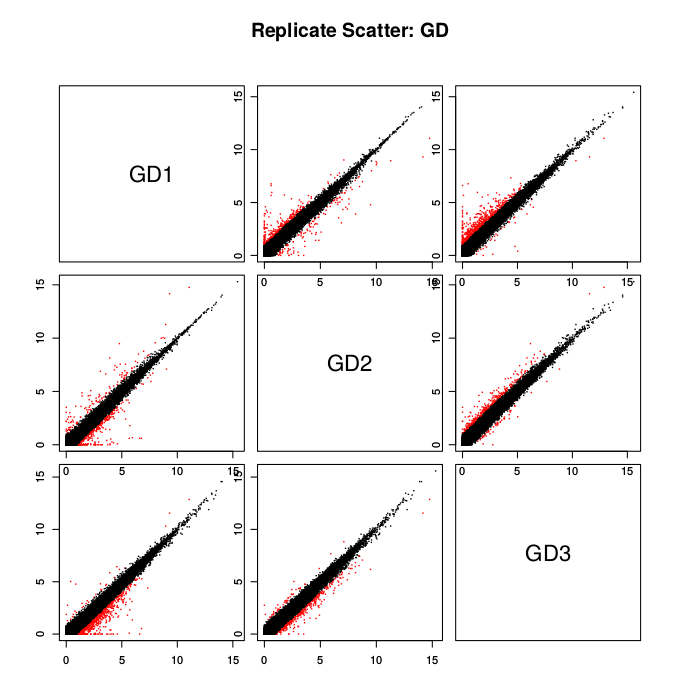


**Supplementary Figure 5:** Pairwise comparisons of replicate log (CPM) values (data points more than 2-fold different are highlighted in red) in the drought stress (GD).


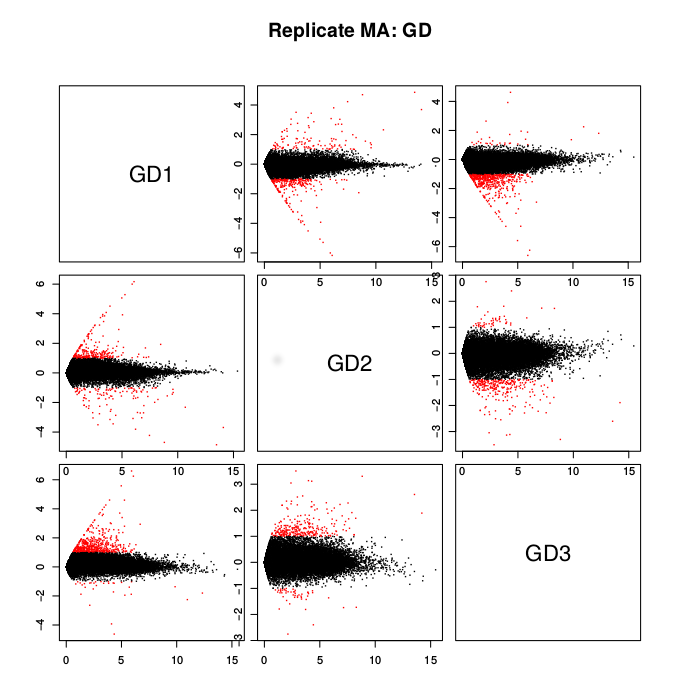


**Supplementary Figure 6:** Pairwise MA plots (x-axis: mean log (CPM), y-axis log(fold_change)) in the drought stress (GD).


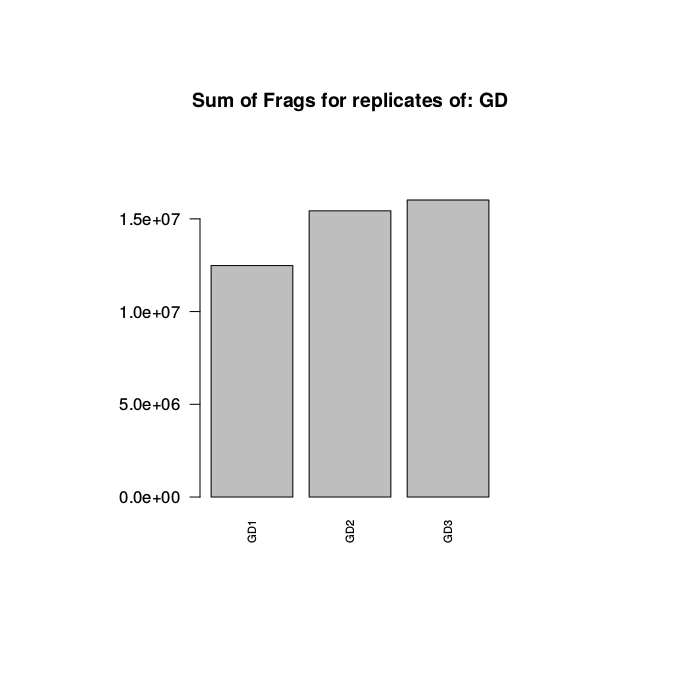


**Supplementary Figure 7:** The total of mapped putative genes in the control (GD).


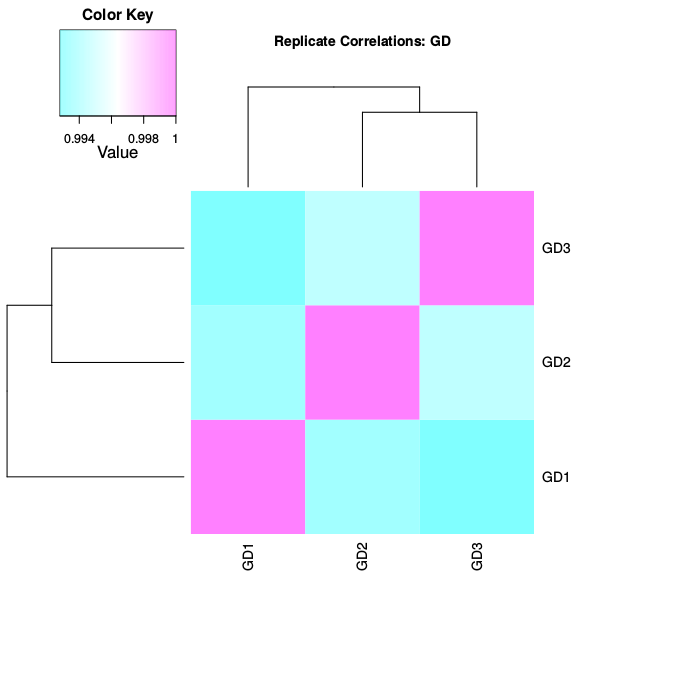


**Supplementary Figure 8:** The heatmap of Pearson correlation replication in the control (GD).


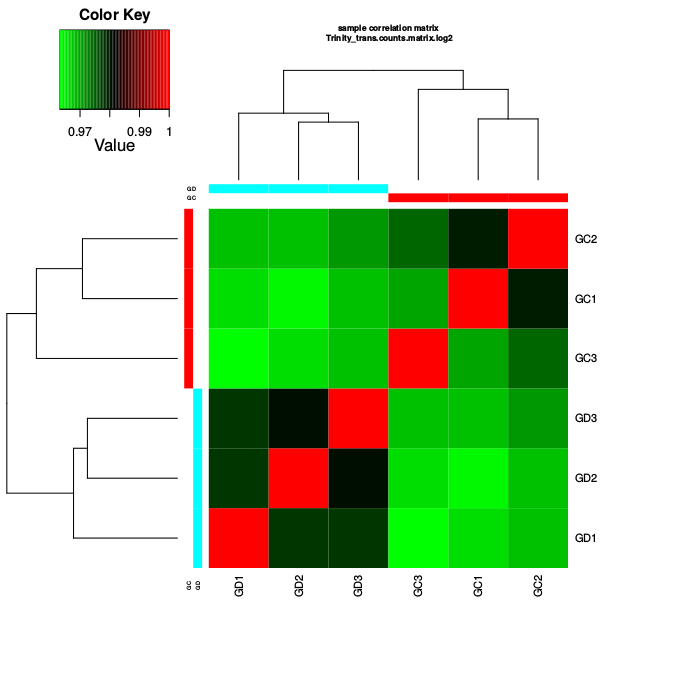


**Supplementary Figure 9:** The correlation matrix between the drought stress (GD) and the control (GC).


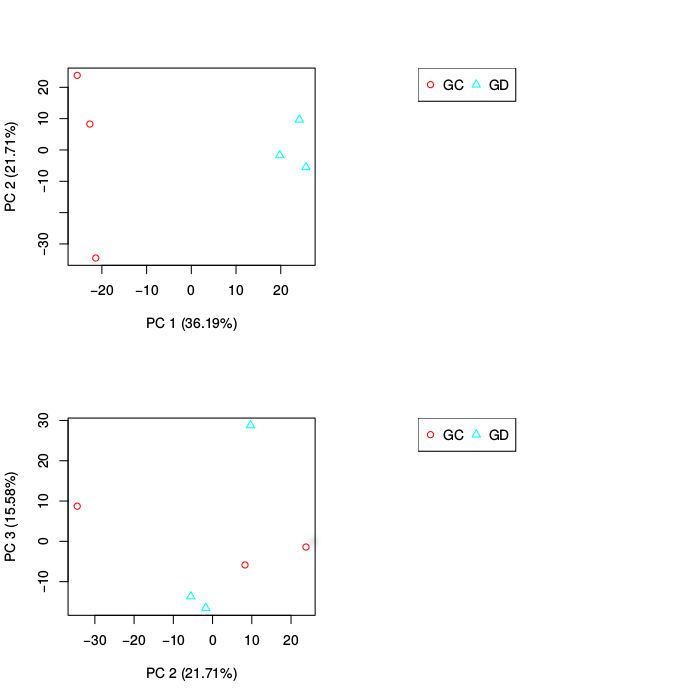


**Supplementary Figure 10:** Principal component analysis (PCA) of drought stress (GD and )the control (GC).


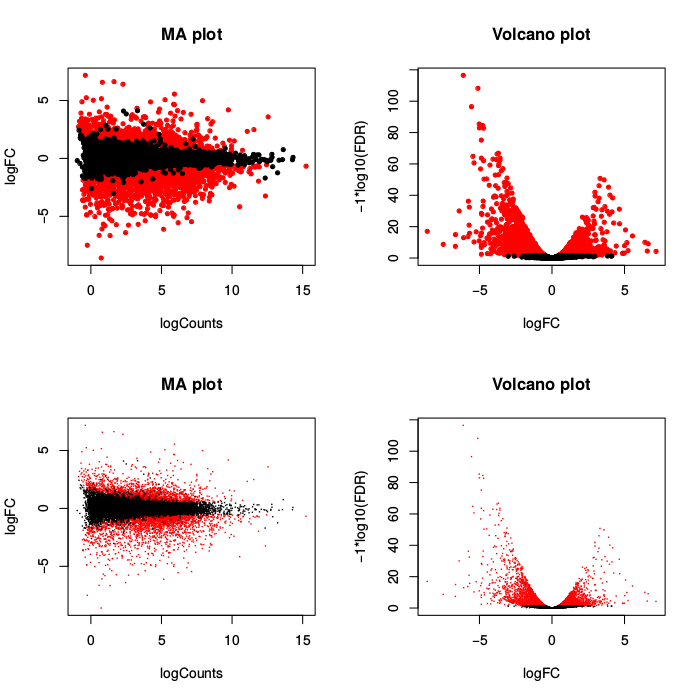


**Supplementary Figure 11:** Distribution of genes according to fold-change (FC), counts and FDR. Significantly DEGs as classified by EdgeR (red) typically show relatively high read counts associated with low expressed genes.


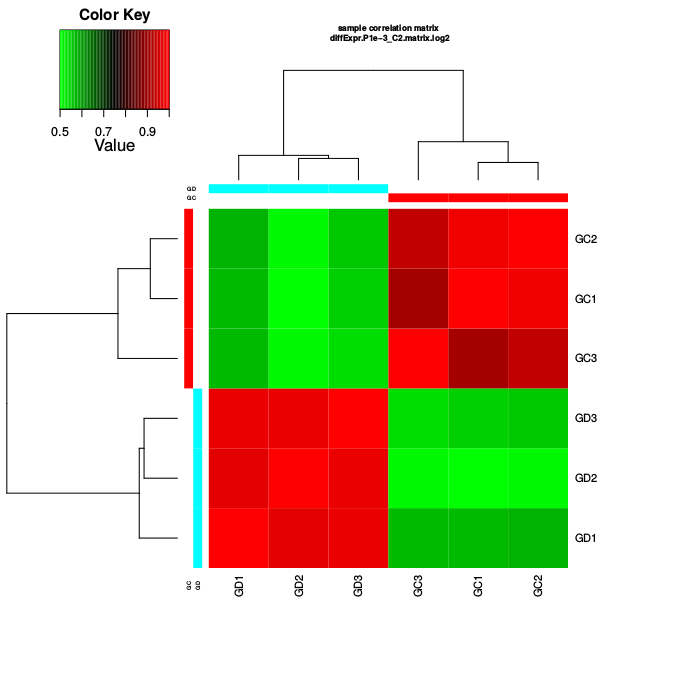


**Supplementary Figure 12:** The clustered heatmap illustrating the correlation matrix of DEGs between the control (GC) and drought stress (GD) at FC of 4 and p-value of 0.001.


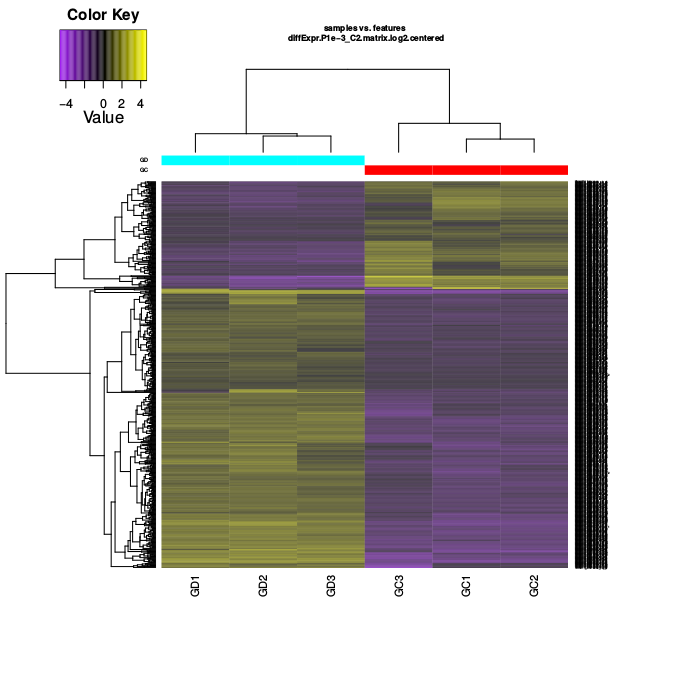


**Supplementary Figure 13:** The DEGs clustered heatmap of control (GC) via drought stress (GD) at FC of 4 and p-value of 0.001.


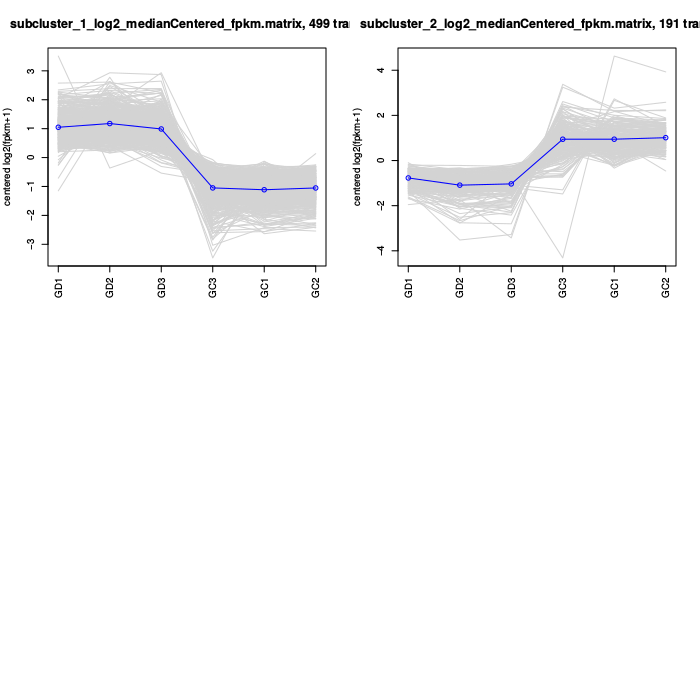


**Supplementary Figure 14:** The expression modalities for the DEGs in each cluster under the control and drought stress. The mean expression profile for that cluster (blue) and gene plots (gray).


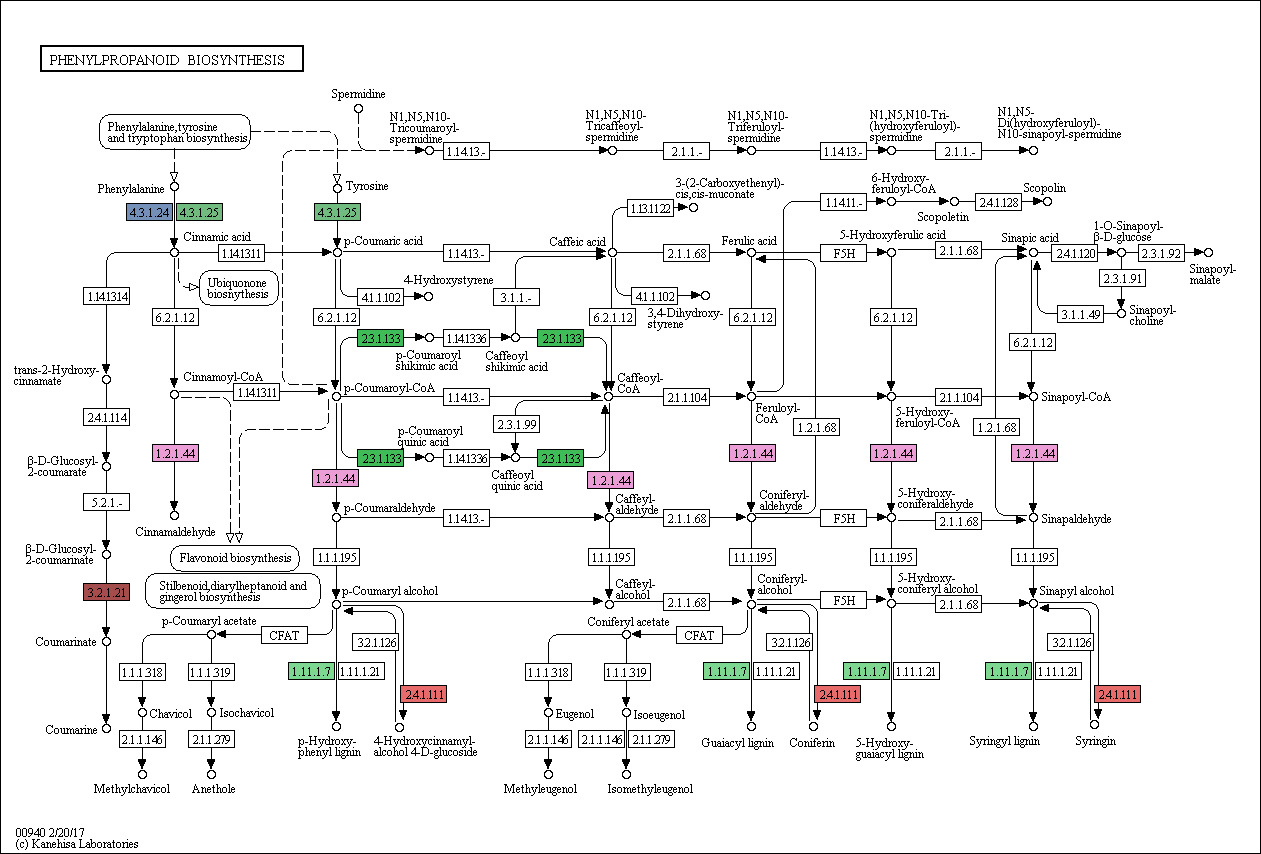


**Supplementary Figure 15:** The pathway of Phenylpropanoid biosynthesis upregulated under drought stress.

**Supplementary table 1:** Values of Principal component analysis for the biological replicates of drought stress (GD) and control (GC).

|  | PC1 | PC2 | PC3 | PC4 | PC5 |
| --- | --- | --- | --- | --- | --- |
| GC1 | -25.52 | 23.82 | -1.41 | 17.45 | 10.31 |
| GC2 | -22.68 | 8.27 | -5.86 | -25.58 | -9.54 |
| GC3 | -21.38 | -34.50 | 8.72 | 5.81 | 1.35 |
| GD1 | 24.17 | 9.67 | 28.78 | -2.55 | -3.37 |
| GD2 | 25.65 | -5.53 | -13.68 | -7.70 | 22.51 |
| GD3 | 19.77 | -1.73 | -16.55 | 12.56 | -21.26 |

**Supplementary table 2:** Values of Pearson correlation between the biological replicates of drought stress (GD) and control (GC).

|  | GC1 | GC2 | GC3 | GD1 | GD2 | GD3 |
| --- | --- | --- | --- | --- | --- | --- |
| GC1 | 1 |  |  |  |  |  |
| GC2 | 0.98 | 1 |  |  |  |  |
| GC3 | 0.97 | 0.97 | 1 |  |  |  |
| GD1 | 0.97 | 0.97 | 0.96 | 1 |  |  |
| GD2 | 0.96 | 0.97 | 0.97 | 0.98 | 1 |  |
| GD3 | 0.97 | 0.97 | 0.97 | 0.98 | 0.98 | 1 |

**Supplementary table 3:** values of Pearson correlation matrix between the biological replicates of drought stress (GD) and control (GC) based on the set of DEGs.

|  | GC1 | GC2 | GC3 | GD1 | GD2 | GD3 |
| --- | --- | --- | --- | --- | --- | --- |
| GC1 | 1.00 |  |  |  |  |  |
| GC2 | 0.98 | 1.00 |  |  |  |  |
| GC3 | 0.91 | 0.94 | 1.00 |  |  |  |
| GD1 | 0.57 | 0.58 | 0.57 | 1.00 |  |  |
| GD2 | 0.50 | 0.51 | 0.51 | 0.97 | 1.00 |  |
| GD3 | 0.55 | 0.56 | 0.54 | 0.98 | 0.98 | 1.00 |

**Supplementary table 4:** List of upregulated KEGG pathways responsive to drought stress.

| Pathway | Pathway ID | #Enzs in Pathway | #Seqs of Enzyme |
| --- | --- | --- | --- |
| Biosynthesis of antibiotics | map01130 | 11 | 11 |
| Starch and sucrose metabolism | map00500 | 8 | 11 |
| Phenylpropanoid biosynthesis | map00940 | 7 | 12 |
| Glycerolipid metabolism | map00561 | 6 | 9 |
| Terpenoid backbone biosynthesis | map00900 | 6 | 6 |
| Pyruvate metabolism | map00620 | 5 | 5 |
| Arginine and proline metabolism | map00330 | 4 | 4 |
| Diterpenoid biosynthesis | map00904 | 4 | 5 |
| Glycerophospholipid metabolism | map00564 | 4 | 8 |
| Carbon fixation in photosynthetic organisms | map00710 | 4 | 4 |
| Glycolysis / Gluconeogenesis | map00010 | 3 | 3 |
| Carotenoid biosynthesis | map00906 | 3 | 10 |
| Phenylalanine metabolism | map00360 | 3 | 5 |
| Tyrosine metabolism | map00350 | 3 | 3 |
| Inositol phosphate metabolism | map00562 | 3 | 4 |
| beta-Alanine metabolism | map00410 | 3 | 3 |
| Fatty acid degradation | map00071 | 3 | 3 |
| Alanine, aspartate and glutamate metabolism | map00250 | 3 | 3 |
| Fatty acid biosynthesis | map00061 | 3 | 3 |
| Sulfur metabolism | map00920 | 3 | 4 |
| Purine metabolism | map00230 | 3 | 5 |
| Butanoate metabolism | map00650 | 3 | 3 |
| Tryptophan metabolism | map00380 | 2 | 2 |
| alpha-Linolenic acid metabolism | map00592 | 2 | 2 |
| Galactose metabolism | map00052 | 2 | 2 |
| Biosynthesis of unsaturated fatty acids | map01040 | 2 | 2 |
| Flavonoid biosynthesis | map00941 | 2 | 5 |
| Histidine metabolism | map00340 | 2 | 2 |
| Phosphatidylinositol signaling system | map04070 | 2 | 3 |
| Drug metabolism - other enzymes | map00983 | 2 | 4 |
| T cell receptor signaling pathway | map04660 | 2 | 4 |
| Porphyrin and chlorophyll metabolism | map00860 | 2 | 2 |
| Isoquinoline alkaloid biosynthesis | map00950 | 2 | 2 |
| Nicotinate and nicotinamide metabolism | map00760 | 2 | 2 |
| Drug metabolism - cytochrome P450 | map00982 | 2 | 3 |
| Cutin, suberine and wax biosynthesis | map00073 | 2 | 2 |
| Steroid biosynthesis | map00100 | 2 | 2 |
| Carbapenem biosynthesis | map00332 | 2 | 2 |
| Other glycan degradation | map00511 | 2 | 2 |
| Glutathione metabolism | map00480 | 1 | 2 |
| Cyanoamino acid metabolism | map00460 | 1 | 1 |
| Stilbenoid, diarylheptanoid and gingerol biosynthesis | map00945 | 1 | 4 |
| Glycosphingolipid biosynthesis - ganglio series | map00604 | 1 | 1 |
| Valine, leucine and isoleucine degradation | map00280 | 1 | 1 |
| Amino sugar and nucleotide sugar metabolism | map00520 | 1 | 1 |
| Metabolism of xenobiotics by cytochrome P450 | map00980 | 1 | 2 |
| mTOR signaling pathway | map04150 | 1 | 2 |
| Sphingolipid metabolism | map00600 | 1 | 1 |
| Aminobenzoate degradation | map00627 | 1 | 10 |
| Tropane, piperidine and pyridine alkaloid biosynthesis | map00960 | 1 | 1 |
| Taurine and hypotaurine metabolism | map00430 | 1 | 1 |
| Cysteine and methionine metabolism | map00270 | 1 | 1 |
| Biosynthesis of siderophore group nonribosomal peptides | map01053 | 1 | 1 |
| Glycine, serine and threonine metabolism | map00260 | 1 | 1 |
| Flavone and flavonol biosynthesis | map00944 | 1 | 1 |
| Th1 and Th2 cell differentiation | map04658 | 1 | 3 |
| Glycosphingolipid biosynthesis - globo and isoglobo series | map00603 | 1 | 1 |
| Sesquiterpenoid and triterpenoid biosynthesis | map00909 | 1 | 1 |
| Indole alkaloid biosynthesis | map00901 | 1 | 2 |
| Lysine degradation | map00310 | 1 | 1 |
| Biotin metabolism | map00780 | 1 | 1 |
| Various types of N-glycan biosynthesis | map00513 | 1 | 1 |
| Biosynthesis of terpenoids and steroids | map01062 | 1 | 1 |
| Vitamin B6 metabolism | map00750 | 1 | 1 |
| Ether lipid metabolism | map00565 | 1 | 2 |
| Zeatin biosynthesis | map00908 | 1 | 3 |
| Nitrogen metabolism | map00910 | 1 | 1 |
| Pentose and glucuronate interconversions | map00040 | 1 | 1 |
| Chloroalkane and chloroalkene degradation | map00625 | 1 | 1 |
| Thiamine metabolism | map00730 | 1 | 3 |
| Carbon fixation pathways in prokaryotes | map00720 | 1 | 1 |
| Glycosaminoglycan degradation | map00531 | 1 | 1 |
| Ascorbate and aldarate metabolism | map00053 | 1 | 1 |
| Streptomycin biosynthesis | map00521 | 1 | 1 |
| Pentose phosphate pathway | map00030 | 1 | 1 |
| Limonene and pinene degradation | map00903 | 1 | 1 |
| Insect hormone biosynthesis | map00981 | 1 | 1 |

**Supplementary table 5:** Best hit of upregulated transcription factors (TFs) in Arabidopsis thaliana.

| **TF ID** | **Family** | **Best hit in A. thaliana** | **Blast e-value** | **Description for the best hit** |
| --- | --- | --- | --- | --- |
| TRINITY_DN13195_c0_g1_i1 | MYB_related | AT5G17300.1 | 5.00E-63 | MYB_related family protein |
| TRINITY_DN13771_c0_g1_i5 | MYB_related | AT3G09600.1 | 1.00E-131 | MYB_related family protein |
| TRINITY_DN15622_c0_g1_i1 | MYB_related | AT1G18330.1 | 6.00E-61 | MYB_related family protein |
| TRINITY_DN16638_c0_g1_i2 | MYB_related | AT1G01060.4 | 1.00E-115 | MYB_related family protein |
| TRINITY_DN16638_c0_g2_i2 | MYB_related | AT1G01060.3 | 1.00E-141 | MYB_related family protein |
| TRINITY_DN5739_c0_g1_i1 | MYB_related | AT3G47600.1 | 8.00E-52 | myb domain protein 94 |
| TRINITY_DN1140_c0_g1_i1 | MYB | AT1G06180.1 | 8.00E-63 | myb domain protein 13 |
| TRINITY_DN12560_c0_g1_i1 | MYB | AT4G37260.1 | 2.00E-97 | myb domain protein 73 |
| TRINITY_DN19324_c0_g12_i1 | MYB | AT4G37260.1 | 2.00E-96 | myb domain protein 73 |
| TRINITY_DN20429_c0_g1_i1 | MYB | AT3G50060.1 | \| 2.00E-58 \| \| --- \| | myb domain protein 77 |
| TRINITY_DN14153_c1_g1_i2 | ERF | AT5G07580.1 | 7.00E-39 | ERF family protein |
| TRINITY_DN15604_c0_g1_i3 | ERF | AT4G13040.1 | 5.00E-44 | ERF family protein |
| TRINITY_DN5802_c0_g1_i1 | ERF | AT5G11590.1 | 2.00E-34 | ERF family protein |
| TRINITY_DN15311_c0_g1_i7 | bHLH | AT4G37850.1 | 6.00E-45 | bHLH family protein |
| TRINITY_DN8979_c0_g1_i1 | bHLH | AT4G37850.1 | 3.00E-56 | bHLH family protein |
| TRINITY_DN20738_c0_g1_i1 | C2H2 | AT5G03150.1 | 1.00E-97 | C2H2-like zinc finger protein |
| TRINITY_DN41174_c0_g1_i1 | C2H2 | AT2G28200.1 | 2.00E-54 | C2H2 family protein |
| TRINITY_DN15427_c0_g1_i4 | DBB | AT1G06040.1 | 2.00E-96 | DBB family protein |
| TRINITY_DN15633_c0_g1_i6 | DBB | AT4G38960.1 | 2.00E-75 | DBB family protein |
| TRINITY_DN15368_c0_g5_i2 | Dof | AT5G39660.1 | 4.00E-84 | cycling DOF factor 2 |
| TRINITY_DN15368_c0_g8_i1 | Dof | AT1G28310.2 | 4.00E-32 | Dof family protein |
| TRINITY_DN12402_c0_g1_i2 | NAC | AT3G44350.2 | 2.00E-90 | NAC domain containing protein 61 |
| TRINITY_DN8386_c0_g1_i1 | NAC | AT1G61110.1 | 1.00E-112 | NAC domain containing protein 25 |
| TRINITY_DN12838_c0_g2_i1 | AP2 | AT1G16060.2 | 4.00E-84 | ARIA-interacting double AP2 domain protein |
| TRINITY_DN10419_c0_g1_i1 | C3H | AT4G29190.1 | 1.00E-145 | C3H family protein |
| TRINITY_DN13713_c0_g1_i2 | HD-ZIP | AT2G46680.1 | 4.00E-70 | homeobox 7 |
| TRINITY_DN5256_c0_g1_i1 | LBD | AT3G02550.1 | 1.00E-73 | LOB domain-containing protein 41 |
| TRINITY_DN17596_c0_g1_i25 | NF-YA | AT3G20910.1 | 2.00E-51 | nuclear factor Y, subunit A9 |
| TRINITY_DN16836_c0_g1_i4 | Nin-like | AT1G20640.2 | 0 | Nin-like family protein |
| TRINITY_DN19592_c0_g6_i2 | SRS | AT5G12330.1 | 5.00E-50 | Lateral root primordium (LRP) protein-related |

**Supplementary table 6:** List of downregulated KEGG pathways responsive to drought stress.

| Pathway | Pathway ID | #Enzs in Pathway | #Seqs of Enzyme |
| --- | --- | --- | --- |
| Biosynthesis of antibiotics | map01130 | 2 | 2 |
| alpha-Linolenic acid metabolism | map00592 | 1 | 1 |
| Arachidonic acid metabolism | map00590 | 1 | 1 |
| Cysteine and methionine metabolism | map00270 | 1 | 1 |
| Drug metabolism - other enzymes | map00983 | 1 | 1 |
| Ether lipid metabolism | map00565 | 1 | 1 |
| Glycerophospholipid metabolism | map00564 | 1 | 1 |
| Glycine, serine and threonine metabolism | map00260 | 1 | 1 |
| Glyoxylate and dicarboxylate metabolism | map00630 | 1 | 1 |
| Linoleic acid metabolism | map00591 | 1 | 1 |
| Other glycan degradation | map00511 | 1 | 1 |
| Pentose and glucuronate interconversions | map00040 | 1 | 1 |
| Phenylalanine, tyrosine and tryptophan biosynthesis | map00400 | 1 | 1 |
| Starch and sucrose metabolism | map00500 | 1 | 1 |
| Sulfur metabolism | map00920 | 1 | 1 |

**Supplementary table 7:** Best hit of downregulated transcription factors (TFs) in Arabidopsis thaliana.

| **TF ID** | **Family** | **Best hit in A. thaliana** | **Blast e-value** | **Description for the best hit** |
| --- | --- | --- | --- | --- |
| TRINITY_DN15732_c0_g1_i2 | bHLH | AT3G24140.1 | 1.00E-19 | bHLH family protein |
| TRINITY_DN24577_c0_g1_i1 | bHLH | AT3G50330.1 | 3.00E-49 | bHLH family protein |
| TRINITY_DN41892_c0_g1_i1 | bHLH | AT2G28160.1 | 1.00E-101 | FER-like regulator of iron uptake |
| TRINITY_DN9730_c0_g2_i2 | bHLH | AT4G37850.1 | 9.00E-51 | bHLH family protein |
| TRINITY_DN15483_c0_g1_i6 | CO-like | AT2G33500.2 | 7.00E-74 | B-box type zinc finger protein with CCT domain |
| TRINITY_DN15628_c0_g1_i5 | CO-like | AT3G07650.2 | 1.00E-138 | CONSTANS-like 9 |
| TRINITY_DN10560_c0_g1_i5 | G2-like | AT3G46640.3 | 2.00E-94 | G2-like family protein |
| TRINITY_DN14273_c0_g1_i1 | G2-like | AT5G06800.2 | 4.00E-31 | G2-like family protein |
| TRINITY_DN11229_c0_g1_i3 | MYB | AT5G49330.1 | 9.00E-78 | myb domain protein 111 |
| TRINITY_DN7364_c0_g1_i1 | MYB | AT1G22640.1 | 1.00E-65 | myb domain protein 3 |
| TRINITY_DN12840_c0_g1_i1 | NAC | AT5G61430.1 | 1.00E-112 | NAC domain containing protein 100 |
| TRINITY_DN18343_c1_g6_i1 | TCP | AT1G53230.1 | 2.00E-85 | TEOSINTE BRANCHED 1, cycloidea and PCF transcription factor 3 |
